# Basal-Supported Oral Therapy with Sitagliptin Counteracts Rebound Hyperglycemia Caused by GLP-1 Tachyphylaxis

**DOI:** 10.1155/2014/927317

**Published:** 2014-03-11

**Authors:** Shu Meguro, Toshihide Kawai, Tomohiro Matsuhashi, Motoaki Sano, Keiichi Fukuda, Hiroshi Itoh, Yoshihiko Suzuki

**Affiliations:** ^1^Department of Internal Medicine, Division of Endocrinology, Metabolism and Nephrology, School of Medicine, Keio University, 35 Shinanomachi, Shinjuku-ku, Tokyo 160-8582, Japan; ^2^Department of Internal Medicine, Division of Cardiology, School of Medicine, Keio University, 35 Shinanomachi, Shinjuku-ku, Tokyo 160-8582, Japan; ^3^Hanzomon Diabetes City Atlas Clinic, 5-3-9 Ichiban-cho, Chiyoda-ku, Tokyo 102-0082, Japan; ^4^Institute of Gerontology, Nippon Medical School, 1-396 Kosugi-cho, Nakahara-ku, Kawasaki, Tokyo 211-8533, Japan

## Abstract

*Introduction*. Treatment with a glucagon-like peptide 1 (GLP-1) analog fails in some patients due to rebound hyperglycemia caused by tachyphylaxis (GLP-1 tachyphylaxis). We investigated the efficacy of basal-supported oral therapy (BOT) with insulin glargine and sitagliptin for counteracting GLP-1 tachyphylaxis. *Materials and Methods*. The subjects were 12 men and 3 women aged 59.9 ± 10.0 years who had been treated with GLP-1 analogs. All of them had developed rebound hyperglycemia caused by GLP-1 tachyphylaxis. Their GLP-1 analog-based therapy was switched to BOT with insulin glargine plus sitagliptin and other medications. The primary outcomes were whether switching of therapy was associated with a change of hemoglobin A_1c_ (HbA_1c_) and whether weight gain occurred. *Results*. Baseline HbA_1c_ was 8.0 ± 0.9%. It decreased to 7.3 ± 0.9% at 3 months after switching (*P* < 0.01) and to 7.2 ± 0.9% at 4 months (*P* < 0.05). Weight gain was 1.1 kg after 1 month (*P* < 0.01) and 2.3 kg after 5 months (*P* < 0.01). *Conclusion*. Switching to BOT with insulin glargine and sitagliptin improved glycemic control. The significant decrease of HbA_1c_ demonstrated that this combination can counteract deterioration of glycemic control due to rebound hyperglycemia secondary to GLP-1 tachyphylaxis. However, weight gain remains a problem.

## 1. Introduction

Glucagon-like peptide 1 (GLP-1) analogs such as liraglutide or exenatide have been available for 3 years in Japan. Several studies have shown that the acute anorectic effect of liraglutide and exenatide is identical in the acute phase, but patients using liraglutide tend to show GLP-1 tachyphylaxis over the long term so that gastric emptying is not delayed, which could lead to the recurrence of overeating and deterioration of glycemic control [1]. Although it is less marked, GLP-1 tachyphylaxis also occurs with exenatide [2]. Interestingly, when the delay of gastric emptying by GLP-1 is antagonized, the glucose-lowering effect of this incretin is largely abolished [3]. Therefore, many patients in Japan have developed “rebound hyperglycemia caused by GLP-1 tachyphylaxis” (RHGT). When treatment with GLP-1 analogs fails in some patients due to RHGT, the subsequent therapeutic options for these patients are limited.

In Japan, no reports have been published about a solution for the problem of RHGT. Sitagliptin has a hypoglycemic effect due to its incretin activity while insulin glargine has a different mechanism of action as basal therapy [4]; so the glucose-lowering effects of sitagliptin and insulin glargine could be complementary. We hypothesized that basal-supported oral therapy (BOT) with oral hypoglycemic agents (OHA), insulin glargine, and sitagliptin, that is, BOT (OHA + glargine + sitagliptin), had the potential to counteract RHGT. To investigate this hypothesis, we studied patients who had experienced RHGT with relatively poor glycemic control for at least 3 months.

## 2. Materials and Methods

### 2.1. Subjects

We analyzed subjects with type 2 diabetes mellitus who had been treated with GLP-1 analogs for at least 3 months and had developed GLP-1 tachyphylaxis. Because of rebound elevation of hemoglobin A_1c_ (HbA_1c_) caused by GLP-1 tachyphylaxis, we switched their treatment to BOT (OHA + glargine + sitagliptin). We excluded the following patients from this study: (1) patients receiving treatment with insulin formulations other than once daily insulin glargine, (2) patients using oral dipeptidyl peptidase-4 (DPP-4) inhibitors other than sitagliptin, (3) patients on steroid therapy, (4) patients who were not indicated for oral medications such as those with severe infection, scheduled major surgery, or serious trauma, and (5) pregnant or nursing women and women who were possibly pregnant. We enrolled 15 subjects and followed them for 5 months before analyzing the data.

All of the subjects were attending Hanzomon Diabetes City Atlas Clinic. They were all Japanese, had been prescribed adequate diet/exercise therapy by specialists and nutritionists, and received other appropriate treatment depending on their medical condition.

This study was approved by the institutional review board of Hanzomon Diabetes City Atlas Clinic and was conducted in accordance with the principles of the Declaration of Helsinki. All subjects were given an explanation of this study and provided informed consent to participation, including informed consent for monthly blood tests.

### 2.2. Treatment

At baseline, adjustment of therapy was done by diabetologists. Throughout the 5-month observation period, the doses of OHA including sitagliptin, dose of insulin glargine, and timing of glargine injection were adjusted carefully according to the patient's lifestyle, and daily glucose excursion was assessed by self-monitoring of blood glucose (SMBG). Changes of OHA dosage were not allowed in general. However, if there was a risk of progressive hypoglycemia in patients using sulfonylureas (SU) or metformin, dose titration was allowed. To improve glycemic control, the dose of insulin glargine was adjusted. Insulin was reduced, if a patient had 2 or more SMBG readings ≤4.4 mmol/L (≤80 mg/dL).

Sitagliptin was started at 100 mg/day, because all of the subjects had already been on incretin therapy. Dosage could be reduced if the attending doctor judged that there was a risk of hypoglycemia, with adjustment being done individually according to each patient's condition to prevent severe hypoglycemia. For example, if a patient experienced hypoglycemia at night, he/she was instructed to inject insulin glargine in the morning, while patients with daytime hypoglycemia were injected glargine in the morning and their SU agent was omitted. If there was a risk of hypoglycemia in patients with concomitant SU therapy, reduction of the SU dose was allowed. All dose modifications were conducted under the direction of diabetologists.

Blood samples were taken to measure fasting plasma glucose but were not analyzed. Because some of the patients on BOT (OHA + glargine + sitagliptin) were likely to develop hypoglycemia at home in the early morning, it was difficult to obtain a proper fasting blood sample at the outpatient department since the subjects often ingested glucose or had breakfast in the early morning [5]. Therefore, not only fasting plasma glucose but also lipid parameters were not analyzed. Only HbA_1c_ and weight were assessed as the primary outcomes in this study. HbA_1c_ and weight were measured in all patients at baseline prior to switching to BOT (OHA + glargine + sitagliptin), as well as at 1 month, 2 months, 3 months, 4 months, and 5 months after switching. The incidence of severe hypoglycemia during the treatment period was also evaluated. HbA_1c_ was measured with an HLC-723G7 (Tosoh Co., Tokyo, Japan).

To investigate nephropathy, spot urine was collected. An albumin/creatinine ratio ≤30 mg/gCr was defined as normal, while a ratio ≥300 mg/gCr was defined as macroalbuminuria. To assess peripheral neuropathy, the posterior tibial nerve conduction velocity was measured and a velocity <40 m/s was defined as positive for neuropathy.

### 2.3. Statistical Analysis

In all patients, the changes of HbA_1c_, body weight, and body mass index (BMI) during the 5-month study period were evaluated by the paired* t*-test and also by the Wilcoxon test. For all analyses, *P* < 0.05 was taken to indicate statistical significance. Results are expressed as the mean ± standard deviation.

## 3. Results

### 3.1. Patient's Characteristics

Patient's characteristics are demonstrated in Table 1. The subjects consisted of 12 men and 3 women. Their mean age was 59.9 ± 10.0 years and the mean duration of diabetes was 14.9 ± 9.2 years. All subjects were switched to treatment with insulin glargine, sitagliptin, and glimepiride at baseline. Differences of their treatment between baseline and after 5 months are listed in Table 2. The previous GLP-1 analog was liraglutide in 9 subjects and exenatide in 6 subjects. Among the 6 subjects, 3 received liraglutide treatment first and then changed to exenatide therapy, because they did not reach enough glycemic control.

As for diabetic complications, 5 subjects had background retinopathy and 4 subjects had proliferative retinopathy, while the others did not show any signs of retinopathy. Two subjects had microalbuminuria and 2 subjects had macroalbuminuria, while the other subjects were normal on urinalysis. Four subjects had peripheral neuropathy.

### 3.2. Changes of HbA_1c_

**Figure 1:**
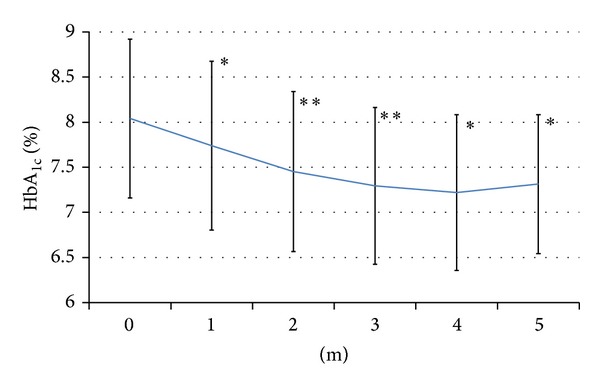
Changes of HbA_1c_. HbA_1c_ decreased significantly compared with baseline. *P < 0.05. **P < 0.01 by the paired* t*-test (versus baseline). Significance was confirmed by Wilcoxon's test.

The baseline HbA_1c_ value was 8.0 ± 0.9%. After switching therapy, HbA_1c_ decreased to 7.7 ± 0.9% in the 1st month (*P* < 0.05), 7.5 ± 0.9% in the 2nd month (*P* < 0.01), 7.3 ± 0.9% in the 3rd month (*P* < 0.01), 7.2 ± 0.9% in the 4th month (*P* < 0.05), and 7.3 ± 0.8% in the 5th month (*P* < 0.05). The changes of HbA_1c_ are displayed in Figure 1.

### 3.3. Changes of Body Weight

**Figure 2:**
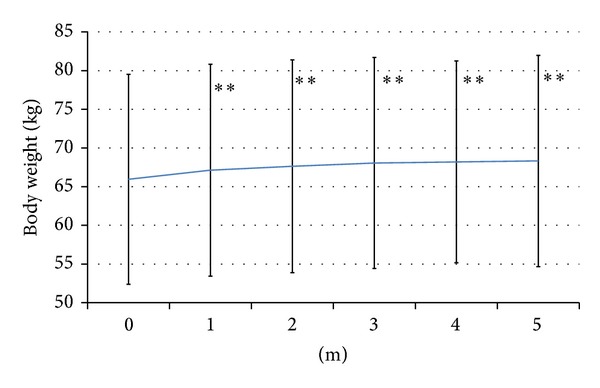
Changes of body weight. Weight gain occurred from 1 month after switching therapy. **P < 0.01.

The body weight was 66.0 ± 13.6 kg at baseline. After switching therapy, weight increased to 67.1 ± 13.7 kg in the 1st month (*P* < 0.01), 67.6 ± 13.8 kg in the 2nd month (*P* < 0.01), 68.1 ± 13.6 kg in the 3rd month (*P* < 0.01), 68.2 ± 13.1 kg in the 4th month (*P* < 0.01), and 68.3 ± 13.7 kg in the 5th month (*P* < 0.01). Changes of body weight are presented in Figure 2.

### 3.4. Changes of BMI

**Figure 3:**
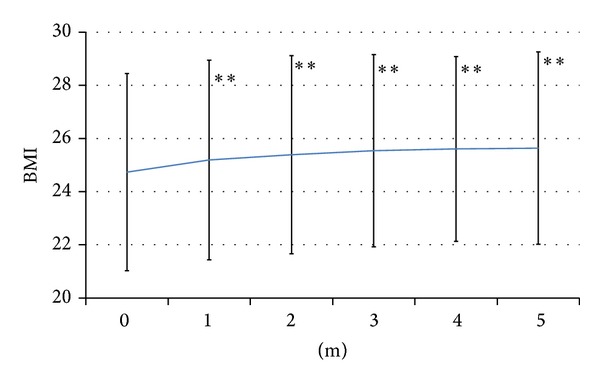
Changes of body mass index. Weight gain was not from 1 month after switching therapy. **P < 0.01.

The baseline value of the BMI was 24.4 ± 3.9. After switching, BMI increased to 24.9 ± 3.9 in the 1st month (*P* < 0.01), 25.0 ± 3.9 in the 2nd month (*P* < 0.01), 25.2 ± 3.7 in the 3rd month (*P* < 0.01), 25.3 ± 3.6 in the 4th month (*P* < 0.01), and 25.3 ± 3.8 in the 5th month (*P* < 0.01). Changes of BMI are presented in Figure 3.

### 3.5. Insulin Dosage

Between baseline and the 5th month, the daily dose of insulin decreased from 11.7 ± 4.5 U/day to 10.4 ± 3.6 U/day.

### 3.6. Oral Hypoglycemic Agents

Fourteen out of 15 subjects started sitagliptin at 100 mg/day, while 1 subject received 25 mg/day. During the 5-month study period, the dose of sitagliptin was not changed. All subjects continued to take glimepiride for 5 months. The mean dose of glimepiride was 2.9 ± 1.8 mg/day at baseline and it was slightly reduced to 2.8 ± 1.7 mg/day. No subject used an *α*-glucosidase inhibitor. Eight subjects received metformin. The mean dose was 1,400 mg/day at baseline, which was slightly reduced to 1,325 mg/day after 5 months. Six subjects received pioglitazone and the mean dose was 27.5 ± 11.3 mg/day during the entire study period.

### 3.7. Hypoglycemic Events

There were 14 episodes of mild hypoglycemia, but no episodes of severe hypoglycemia during the study period. The patients with mild hypoglycemia were able to prevent moderate/severe hypoglycemia by compliance with their instructions for additional food intake to manage hypoglycemia [5].

## 4. Discussion

In Japan, the number of patients with RHGT is increasing. When 28 patients received 6 months of liraglutide therapy, Yonemoto et al. reported that 30–50% experienced RHGT [6], while Tanaka et al. reported that 14 out of 19 patients (73%) developed RHGT after 1 year of liraglutide treatment [7]. At our hospitals, 63 out of 93 patients (67%) who started liraglutide had to switch therapy because of RHGT over a 2-year period (unpublished data). Thus, the prevalence of RHGT seems to be higher in Japan than in other countries. Several possibilities for this can be considered. Because the maximum dose of liraglutide that can be used in Japan is set at 50% of the level in the United States (1.8 mg/day) and 75% of that for China (1.2 mg/day), many Japanese diabetologists consider that the dose limit of 0.9 mg/day that only applies in Japan might be too low. Another reason is that combining liraglutide with oral hypoglycemic therapy other than SU agents, such as metformin, pioglitazone, or *α*-glucosidase inhibitors, is not approved in Japan. Therefore, we were obliged to find an alternative regimen that could be employed in Japan. In the present study, BOT (OHA + glargine + sitagliptin) exerted a strong hypoglycemic effect and counteracted the rebound elevation of HbA_1c_ caused by GLP-1 tachyphylaxis. The decline of HbA_1c_ shown in Figure 1 indicates the excellent hypoglycemic effect of BOT with OHA + glargine + sitagliptin.

Because DPP-4 inhibitors promote early intrinsic insulin secretion from the pancreas that ameliorates postprandial hyperglycemia and because basal insulin therapy acts over 24 hours, the combination of these two medications is theoretically suitable for suppressing hyperglycemia throughout the day in patients with type 2 diabetes. DPP-4 inhibitors have been thought to exert their effects mainly through inhibition of GLP-1 degradation. However, a study carried out in a rodent model showed the absence of development of tachyphylaxis following administration of vildagliptin, another DPP-4 inhibitor, for 3 weeks [8]. A recent human study using exendin-9, a GLP-1 receptor inhibitor, showed that inhibition of GLP-1 degradation could explain only about 50% of the effect of DPP-4 inhibitors and that GLP-1-independent mechanisms were also involved in the glucose-lowering effects of this class of drugs [9]. Therefore, we administered insulin and sitagliptin to subjects who fail to respond to GLP-1 analog treatment alone. In fact, the present study confirmed that the glucose-lowering action of sitagliptin and insulin glargine was complementary [10]. It also supported the previous report that improvement of HbA_1c_ in Japanese patients was significantly greater with basal insulin therapy than with twice daily insulin or multiple doses [11]. These results and our findings together suggest that adding sitagliptin to basal insulin therapy is an effective strategy.

In the present study, changes to the doses of concomitant oral antidiabetic agents were allowed if necessary, and the dose of insulin could be titrated. To suppress postprandial hyperglycemia, metformin has been used with sitagliptin for an additive effect and dose uptitration or add-on metformin has been shown to provide substantial and durable improvement of glycemic control while being well tolerated [12]. Among the 15 subjects of this study, 2 needed reduction of the glimepiride dose, 2 needed dose reduction of metformin, one subject needed dose increase of metformin. These dose changes were well tolerated and did not influence the incidence of hypoglycemia.

The main problem with BOT using OHA + glargine + sitagliptin was weight gain (Figures 2 and 3). Weight gain typically occurs with insulin therapy because of improved glycemic control. Relatively high insulin levels achieved with glargine and glimepiride plus addition of sitagliptin might prevent lipolysis and thereby cause weight gain. Other explanations are also possible. For instance, because the anorectic effect of GLP-1 analog therapy was already attenuated by tachyphylaxis, this effect of sitagliptin might have been too weak to prevent the resumption of overeating. Because patients are at risk of hypoglycemia when using insulin glargine or SU agents, they are likely to take precautions to prevent hypoglycemia and this could lead to weight gain. A similar finding was obtained in 2007 by a study which clarified the different effects of a GLP-1 analog and insulin glargine on body weight [13].

Generally, weight gain is the first step towards a state of insulin resistance, while higher insulin doses increase the risk of hypoglycemia, fluid retention, and congestive heart failure. Therefore, we need a way to solve this problem of BOT. Sodium-glucose cotransporter 2 (SGLT2) is a low-affinity, high-capacity transporter that regulates renal glucose reabsorption. It reduces glucose reabsorption and increases glucose excretion by the kidneys and also reduces hyperglycemia in a dose-dependent manner. SGLT2 inhibitors can reduce the weight of patients irrespective of insulin use. In a randomized trial, good glycemic control was obtained with a stable insulin dose and weight loss, without any increase of major hypoglycemic episodes [14]. Schernthaner et al. reported that SGLT2 inhibitors have a longer duration of hypoglycemic activity than DPP-4 inhibitors [15]. Therefore, calorie loss due to increased urinary glucose excretion with an SGLT2 inhibitor might be able to counter weight gain due to BOT with OHA + glargine + sitagliptin in the future [16].

## 5. Conclusion

It is impossible to draw definite conclusions from our study, since a longer-term study in a larger patient population is needed to confirm these findings. However, this study demonstrated that an oral incretin-based treatment strategy employing sitagliptin and insulin glargine may be safe and effective in patients with type 2 diabetes who have developed RHGT. Because pharmacological treatment of type 2 diabetes has been enriched by the availability of incretin therapy in recent years, numerous methods of using incretin analogs have been developed. To solve the problem of RHGT, physicians should make a choice based on clinical characteristics, therapeutic goals, and the patient's preference.

## Figures and Tables

**Table 1:** Baseline characteristics of patients.

Parameters	
*N* (male/female)	15 (12/3)
Duration (years)	14.9 ± 9.2
Age (years)	59.9 ± 10.0
BMI (kg/m^2^)	24.4 ± 3.9
SBP (mmHg)	129.6 ± 12.8
DBP (mmHg)	76.7 ± 14.8
HbA_1c_ (%)	8.0 ± 0.9
LDL-C (mg/dL)	99.7 ± 24.8
Insulin dosages (U)	11.7 ± 4.5

Data are shown as mean ± SD.

BMI: body mass index; SBP: systolic blood pressure; DBP: diastolic blood pressure.

HbA_1c_ is presented as National Glycohemoglobin Standardization Program (NGSP) value.

**Table 2:** Differences of parameters between baseline and after 5 months of treatment.

	Baseline (*n* = 15)	5 months (*n* = 15)
Prior therapy		
Liraglutide (*n*)	9	
Exenatide (*n*)	6	
Study medications		
Insulin glargine (*n*)	15	15
units/day	11.7 ± 4.5	10.4 ± 3.6
Sitagliptin (*n*)	15	15
25 mg/day	1	1
100 mg/day	14	14
Glimepiride (*n*)	15	15
mg/day	2.9 ± 1.8	2.8 ± 1.7
Metformin (*n*)	8	8
mg/day	1400	1325
Pioglitazone (*n*)	6	6
mg/day	27.5 ± 11.3	27.5 ± 11.3
